# Effects of T-Cell Depletion on Allogeneic Hematopoietic Stem Cell Transplantation Outcomes in AML Patients

**DOI:** 10.3390/jcm4030488

**Published:** 2015-03-19

**Authors:** Gabriela Soriano Hobbs, Miguel-Angel Perales

**Affiliations:** 1 Adult Leukemia Service, Massachusetts General Hospital, Boston, MA 02114 USA; E-Mail: ghobbs@partners.org; 2 Harvard Medical School, Boston, MA 02115, USA; 3 Adult Bone Marrow Transplantation Service, Memorial Sloan Kettering Cancer Center, New York, NY 10065, USA; 4 Weill Cornell Medical College, New York, NY 10065, USA

**Keywords:** AML, CD34+ selection, T-cell depletion, graft-*versus*-host disease, hematopoietic stem cell transplantation

## Abstract

Graft *versus* host disease (GVHD) remains one of the leading causes of morbidity and mortality associated with conventional allogeneic hematopoietic stem cell transplantation (HCT). The use of T-cell depletion significantly reduces this complication. Recent prospective and retrospective data suggest that, in patients with AML in first complete remission, CD34+ selected grafts afford overall and relapse-free survival comparable to those observed in recipients of conventional grafts, while significantly decreasing GVHD. In addition, CD34+ selected grafts allow older patients, and those with medical comorbidities or with only HLA-mismatched donors to successfully undergo transplantation. Prospective data are needed to further define which groups of patients with AML are most likely to benefit from CD34+ selected grafts. Here we review the history of T-cell depletion in AML, and techniques used. We then summarize the contemporary literature using CD34+ selection in recipients of matched or partially mismatched donors (7/8 or 8/8 HLA-matched), and provide a summary of the risks and benefits of using T-cell depletion.

## 1. Introduction

Cytogenetic risk stratification in acute myelogenous leukemia (AML) allows clinicians to determine which patients are most likely to benefit from allogeneic hematopoietic stem cell transplantation (HCT), with evidence to support a survival advantage in patients with intermediate or high-risk cytogenetics [1]. In addition to more accurate patient selection based on cytogenetic risk factors, over the past decades, transplantation outcomes have also improved as a result of more accurate patient selection tools, such as the HCT Sorror comorbidity index [2], improvements in HLA matching techniques and supportive care.

Despite these improvements, graft-*versus*-host disease (GVHD) remains a leading cause of post-transplant morbidity and mortality. A variety of T-cell depletion (TCD) techniques have been developed and used over the years in an effort to reduce transplant-related mortality (TRM) due to GVHD (Table 1). In this chapter we will focus on outcomes of HCT using T cell depletion for the treatment of AML in recipients of matched or partially mismatched donors (7/8 or 8/8 HLA-matched), with a primary focus on *ex vivo* CD34+ selection of the graft. Other methods of T-cell depletion will be mentioned for historic context only.

## 2. T-Cell Depletion Techniques

The goal of T-cell depleting a graft is to reduce GVHD while maintaining the graft-*versus*-leukemia or lymphoma (GVL) effect. A variety of TCD techniques have been used with mixed results. When reviewing reports utilizing TCD for transplantation it is critical to determine the following: (1) Which technique is being used? (2) Which cell population is being removed (*i.e.*, T, B, NK or all non-hematopoietic cells) and to what extent? Different techniques lead to both quantitative and qualitative differences in the cells being depleted with important clinical implications; (3) Is post-transplant GVHD prophylaxis utilized? and (4) What is the graft source and what is the degree of HLA matching? All of these factors significantly affect outcomes and therefore must be considered when interpreting published reports.

Early TCD techniques differed in the use of negative *vs.* positive selection. Negative selection can be achieved either through physical methods such as counterflow elutriation [3,4,5] or soybean lectin agglutination (SBA) and sheep red blood cell (sRBC)-rosette depletion (E-rosetting) [6,7,8,9,10], or immunological methods using monoclonal antibodies [5,11,12,13,14,15,16,17,18,19,20,21,22,23,24,25,26,27,28,29]. Monoclonal antibodies can be used with or without complement, or conjugated to toxins. Antibodies vary in their specificities, which can be narrow, such as T10B9 targeting the α/β T cell receptor (TCR), or broad, such as combination of antibodies targeting CD2, CD4 and CD8 [30].

**Table 1:** Results of T-cell depletion (TCD)-PBSCT in patients with acute myelogenous leukemia (AML) ^1^.

Method	Number of Patients	Patients With AML	Donor	Degree of Depletion	GVHD Prophylaxis	Acute GVHD	Graft Failure	EFS/DFS ^2^	OS ^2^	Reference
CD34+	50	29	HLA-MRD	NR	Cyclosporine or Cyclosporine + Steroids	16%	0	DFS 65%	Not reported	[31]
CD34+E−	52	21	HLA-MRD	5 logs	None	8%	0	NR	17% 1 year	[32]
CD34+E−	29	16	HLA-MUD or HLA-MMUD	5 logs	None	9%	3%	57% at 4 years	59% at 4 years	[33]
CD34+	47	47	HLA-MRD	4.9 logs	None	22.7%	0	EFS 63% at 4 years	71% 4 years	[34]
CD3/CD19 depletion	29	16	Haplo	4.4 logs	None	48%	0	35% at 1 year	31% at 241 days	[35]
CD34+E− or CD34+	115	115	HLA-MUD or MRD	NR	None	5%		RFS 58% at 3 years	57% at 3 years	[36]

^1^ Abbreviations: CD34+: CD34-selection; CD34+E−: CD34-selection and E-rosetting; NR: not reported; ^2^ DFS and OS are reported for the entire patient population included in the studies.

Currently, the most common method for T cell depletion relies on positive selection of CD34+ hematopoietic stem cells from the graft [32,33,34,36,37]. CD34+ selection of peripheral blood stem cells (PBSCs) was performed in initial studies with the ISOLEX 300i magnetic cell selection system (Baxter, Deerfield, IL, USA), followed by E-rosetting [32,33,37]. The ISOLEX device is no longer being manufactured, and the most commonly used method in current studies uses immunomagnetic beads with the CliniMACS CD34 Reagent System (Miltenyi Biotech, Gladbach, Germany) for CD34+ selection [31,34,37]. The CliniMACS system can also be used to negatively select grafts through depletion of CD3+ and CD19+ cells or depletion of TCRαβ+ T cells [35,38,39,40,41]. The two CD34+ selection methods differ in the degree of TCD; for example, the CliniMACS CD34 Reagent System can achieve a 5-log reduction in T cells, whereas the ISOLEX 300i system achieves a 3.5-log reduction, requiring additional T-cell depletion through E-rosetting.

The cell dose and graft source also impact outcomes. In recipients of T-cell depleted marrow grafts from HLA-identical donors, the risk of GVHD was shown to increase if the graft contained >1 × 10^5^ T cells/kg [42]. Differences between TCD methods can have a significant impact on clinical outcomes, including the risk of graft failure, GVHD and relapse.

## 3. Outcomes in AML with T-Cell Depletion

One of the primary benefits of allogeneic HCT is derived from the GVL effect, driven by the recognition of tumor cells by donor T cells. Therefore, a concern in the utilization of TCD is the potentially negative impact on relapse resulting from reduced T-cell doses included in the graft. However, it is clear that certain diseases rely more on the GVL effect than others. For example, early studies with TCD in chronic myelogenous leukemia (CML) were associated with a significantly increased risk of relapse. In a retrospective study of 46 patients who received TCD grafts form HLA-identical siblings, relapse at 3-years was significantly higher in the TCD group (62% *vs*. 24%, *p* = 0.0003). However, a significant proportion of these patients were then salvaged with donor lymphocyte infusion (DLI), supporting the role of GVL for this disease [43].

On the other hand, studies utilizing TCD in AML and ALL have shown favorable outcomes in recipients of 7/8 or 8/8 HLA matched related or unrelated donors that are comparable to those seen in unmodified, conventional grafts, calling into question the contribution of GVL in those diseases [34,36,44,45].

## 4. TCD in AML—Early Studies

Studies using TCD done in the 1980’s and 1990’s mostly used bone marrow from sibling donors as the graft source and used a variety of techniques (lectin separation, elutriation, E-rosettes, antibodies against T and NK cells, antibodies with or without complement). These techniques were associated with a 1–2 log reduction in T cells in the graft, and patients were often given post-transplant cyclosporine for additional GVHD prophylaxis. These studies were also associated with increased graft failure [8,46]. The addition of antithymocyte globulin (ATG) and thiotepa to the conditioning regimens, which had traditionally included cyclophosphamide and total body irradiation (TBI) or busulfan, decreased the rate of graft failure and eliminated the need for post transplant GVHD prophylaxis. Favorable results were reported in recipients of HLA-identical donors using this approach along with sequential soybean lectin agglutination and sheep red blood cell-rosette depletion. A study by Papadopoulos *et al.* included 31 patients with AML in CR1 or CR2 and showed a disease-free survival (DFS) at 4 years of 70% with no GVHD or graft rejection [9]. Similar results were reported by Aversa *et al.* using the same regimen in 54 patients with acute leukemia, including 2 with HLA-DR mismatched donors [10]. No GVHD or graft rejection was observed and the event-free survival at 4.9 years was 74% in 30 patients with AML.

These early studies demonstrated the feasibility of TCD, and overcame graft rejection with the utilization of ATG, at the expense of added immunosuppression and ensuing delayed immune recovery.

## 5. TCD in AML—Contemporary Studies

Contemporary studies have utilized two main approaches of TCD by CD34+ selection described above, either the ISOLEX 300i Magnetic Cell Separator followed by sRBC-rosette depletion or, more recently, the Miltenyi CliniMACS CD34 Reagent System. In 2011, Devine *et al.* [34] reported the results of a Blood and Marrow Clinical Trials Network study (BMT CTN 0303) utilizing CD34+ selection with the Miltenyi CliniMACS CD34 Reagent System. The study included 44 patients with AML in CR1 or CR2 (excluding patients with *t*(15;17), and core binding factor leukemia) who received T cell depleted PBSCT from HLA-identical siblings after conditioning with hyperfractionated TBI (HFTBI), thiotepa, cyclophosphamide and ATG. No additional GVHD prophylaxis was given. All patients engrafted; the incidence of aGVHD (grades II–IV) was 22.7% and extensive cGVHD was 6.8% at 2 years. The relapse rate at three years for patients in CR1 was 17.4%, with a DFS for all patients at 6 months of 82% and a 2-year OS of 60%. Rates of infection were comparable to other studies; however EBV reactivation occurred in 18% of patients leading to 1 death from Epstein-Barr Virus (EBV) post-transplant lymphoproliferative disease (PTLD) [36,47,48]. In a second report, the outcomes of patients from the BMT CTN 0303 study were compared to a similar cohort of patients (AML in either CR1 or CR2, PBSCT from HLA-identical siblings) who received a conventional HCT on BMT CTN 0101 study (a study comparing fluconazole with voriconazole as antifungal prophylaxis after HCT) [44]. There were no differences in leukemia relapse (23% *vs.* 27% in the TCD and conventional graft groups, respectively) and 2-year OS (65% *vs.* 59% in the TCD and conventional graft groups, respectively). However rates of GVHD were higher in the conventional graft group (aGVHD 23% *vs.* 39%, *p* = 0.07 and cGVHD 19% *vs.* 50%, *p* < 0.001).

More recently, a retrospective study compared the use of TCD HCT to conventional grafts in patients with AML in CR1 by examining outcomes of 115 patients who received TCD grafts at Memorial Sloan Kettering Cancer Center (MSKCC) with a cohort of 181 patients at MD Anderson Cancer Center (MDACC) [36]. A hundred and seven patients in the MSKCC cohort received PBSC grafts, including 85 that were CD34-selected with the ISOLEX 300i Magnetic Cell Separator followed by sRBC-rosette depletion, and 22 with the Miltenyi CliniMACS CD34 Reagent System. Patients at both centers received myeloablative conditioning (MAC) and both cohorts included recipients of matched related, matched unrelated and mismatched donors. Patients at MSKCC were more likely to be recipients of a mismatched graft (27% *vs.* 14%, *p* < 0.001). Patients at MSKCC did not receive additional GVHD prophylaxis. Patients at MDACC received tacrolimus and mini-methotrexate for GVHD prophylaxis, and ATG for HLA-mismatched donors. There were no significant differences in the rate of relapse at 3 years between groups (18% *vs.* 25%, in the TCD *vs.* conventional grafts, respectively, *p* = 0.3). However, rates of GVHD were significantly lower in the TCD group (5% *vs.* 18% for aGVHD, *p* = 0.005, and 13% *vs.* 53% for cGVHD, *p* < 0.001).

Although contemporary studies that compare outcomes of conventional to TCD grafts are retrospective, the results suggest that TCD transplants offer similar DFS and OS with significantly lower rates of GVHD.

As noted above additional TCD approaches are being investigated beyond CD34 selection [35,38,39,40,41]. The potential advantages of negative selection by depletion of CD3+/CD19+ or TCRαβ+ T cells include the presence of additional cells in the graft such as natural killer (NK) cells or TCRγδ+ T cells, which may play a role in relapse or infection prevention. To date, the published studies of these approaches have been in recipients of haplo-identical grafts and there has been limited data on patients with AML. In a study by Bethge *et al*. [35], EFS was 35% at one year in 16 patients with AML who received a CD3/CD19 depleted transplant from a haplo-identical donor.

Finally, an alternative GVHD prophylaxis approach that will be compared to CD34-selection in an upcoming phase 3 trial (BMT CTN 1301) relies on the use of post-transplant high dose cyclophosphamide after a T-replete bone marrow graft from a matched donor [49,50,51]. In a recently published study using this approach in 138 patients with AML, the 3-year DFS and OS were 43% (95% CI, 35% to 52%) and 53% (95% CI, 45% to 62%), respectively [50]. DFS (48% *vs.* 29% at 3 years) and OS (55% *vs.* 50% at 3 years) were higher in patients with AML in morphologic CR compared to those with active disease. The approach was associated with low rates of grade III to IV acute GVHD (11% at 100 days) and chronic GVHD (13% at 2 years).

## 6. Impact of T-Cell Depletion on Engraftment and Immune Reconstitution

Hematopoietic stem cell transplantation, regardless of donor source and manipulation, is associated with significant and prolonged immunosuppression and risk of severe and fatal infections [48,52], disease relapse and secondary malignancies [20,53]. The use of TCD, whether *in vivo* with agents such as alemtuzumab or ATG, or *ex vivo* with T-cell depletion considerably affects immune recovery. In early studies comparing immune reconstitution in TCD and unmodified grafts the rate of CD3+, CD4+ and CD8+ T cell reconstitution was significantly delayed in TCD recipients, correlating with increased risk of infections, including EBV-PTLD [48,54]. T-cell receptor (TCR) studies using 5′-RACE PCR with deep sequencing have confirmed these findings by showing more rapid recovery of TCR diversity in conventional graft recipients compared to TCD grafts [55]. Lower T-cell levels result from decreased thymic output, which can be quantified via measurements of T-cell receptor excision circles (TRECs). Studies have shown that older patients and recipients of TCD grafts have lower TRECs than unmodified graft recipients; however this difference abates beyond 9 months [56].

ATG plays an important role in TCD by reducing the risk of graft rejection. However it is associated with delayed immune recovery and an increased risk of opportunistic infections (OI), with approximately 15% of the patients in early TCD studies dying from OIs [9,10]. Furthermore, a study of immune reconstitution in patients receiving TCD grafts with or without ATG found delayed immune reconstitution with ATG, which was associated with increased OIs [54]. Although studies of TCD performed without ATG by substituting cyclophosphamide with fludarabine have demonstrated durable engraftment in recipients of matched related donors, there did not appear to be a significant effect on immune recovery or the risk of OIs [32].

It is important to note however that, in addition to age and TCD, the presence of GVHD also significantly hampers immune reconstitution via direct effects on the thymus [57,58,59], as well as the immunosuppressive drugs required for treatment of GVHD [60,61,62,63,64]. Although TCD impacts immune reconstitution leading to higher infection related deaths, GVHD in conventional grafts similarly leads to increased mortality. This is a potential explanation for the similar RFS and OS outcomes observed in patients with AML in the MSKCC/MDACC retrospective study and the BMT CTN study [36,44].

## 7. Strategies to Enhance Immune Recovery Post HCT

HCT affords a curative treatment option to many patients with otherwise incurable malignancies. However, the benefit of this therapy comes at the risk of significant complications, including infection, GVHD, relapse and secondary malignancies [20,48,52,53,65,66]. The rationale for TCD is to mitigate GVHD while preserving the benefit of GVL. TCD and HCT in general, are associated with prolonged immunosuppression. Therefore, strategies to optimize post transplant immunity, enhance GVL, while minimizing infectious complications are needed.

The addition of T cells post transplantation represents one such strategy. In one recent study, 19 pediatric patients (13 transplanted for malignant disease) received CD34+ selected matched unrelated donor (MUD) HCT with CD3+ T cells added back, at a dose of 1.0–2.5 × 10^5^ CD3+/kg, and tacrolimus for GVHD prevention [67]. Rates of aGVHD, cGVHD and extensive cGVHD were 15.8%, 23.3% and 0%, respectively, which are low compared to conventional HCT. All patients on this study had neutrophil engraftment, and infection-related mortality at one year was 5%–6%, showing the feasibility of this approach. This approach has also been used in recipients of CD34-selected haplo-identical grafts [68]. The same group previously reported low rates of acute and chronic GVHD in a retrospective series of 16 patients who received DLI (up to 6 × 10^4^ CD3+/kg) from haplo-identical donors to enhance immune recovery and/or treat infections [69]. One ongoing trial is evaluating the effect of serial DLI post TCD in patients with advanced multiple myeloma (NCT01131169).

Another strategy to boost immune reconstitution post-transplant is the use of Keratinocyte Growth Factor (KGF). KGF has been shown in pre-clinical models to play an important role in T cell homeostasis and immune recovery, as well as in thymic regeneration after radiation injury [70,71,72]. Based on these data, KGF along with sex steroid ablation is being studied in an ongoing phase II clinical trial (NCT01746849).

We recently published the results of a phase I study using recombinant human IL-7 (rhIL-7, CYT107, Cytheris) in recipients of TCD HCT and demonstrated enhanced immune recovery, with significant increases in CD4+ and CD8+ T cells along with increased T cell function, without causing significant GVHD or other serious toxicity [73].

## 8. Benefits of TCD HCT in AML

Although reduced intensity conditioning (RIC) regimens allow older patients to undergo HCT, they are associated with higher relapse rates in AML [74,75]. However, the combined toxicity of GVHD prophylaxis that usually includes a calineurin inhibitor (CNI) and methotrexate with myeloablative conditioning (MAC), makes this approach prohibitive in older patients. Unlike conventional grafts, CD34+ selected grafts do not require post-transplant GVHD prophylaxis, and as a result, older patients can be treated with MAC. In addition, patients with renal insufficiency can also successfully undergo transplantation by avoiding the use of CNIs.

The use of CD34+ selected grafts is associated with significant reductions in both acute and chronic GVHD. In addition to the obvious advantage of lowering GVHD, patients without fully matched donors are also able to undergo transplantation, therefore expanding the pool of potential donors.

Finally, CD34+ selected grafts are an ideal platform for post-transplant immunotherapy with adoptive cell therapy targeting both minimal residual disease and viral reactivation by CMV and EBV, among others [76,77,78,79]. The administration T cells specific for tumor or viral antigens post-transplant has the potential advantage of overcoming any loss of GVL or increased infectious risk associated with TCD without affecting the benefit of reduced GVHD.

## 9. Conclusions

After three decades of investigation, it is reasonable to consider CD34+ selected allografts for patients with AML in CR1 based on prospective data [34], and well conducted retrospective studies [36,44]. These contemporary studies are significantly more homogeneous in their methodology and patient inclusion than prior studies, mostly using the CliniMACS CD34 Reagent System for CD34+ selection, and reporting consistent favorable outcomes for patients with AML in CR. The use of CD34+ selected grafts overcomes the morbidity and mortality associated with GVHD, a significant contributor of transplant-related complications, without compromising the benefit of transplantation and affording the same overall survival as conventional transplantation.

TCD represents an important step in graft manipulation, allowing older patients, and those with comorbidities to successfully undergo transplantation. Ongoing research aims to continue to decrease morbidity and mortality associated with transplantation by improving immune reconstitution and the GVL effect.

As mentioned above, an ongoing national phase 3 trial will compare TCD with the CliniMACS CD34 Reagent System to post-transplant cyclophosphamide [49], and a control arm (tacrolimus and methotrexate) in patients with acute leukemia and MDS who are eligible for a MAC transplant from a matched related or unrelated donor (BMT CTN 1301).
